# Expression of Immune Checkpoint Receptors in Placentae With Infectious and Non-Infectious Chronic Villitis

**DOI:** 10.3389/fimmu.2021.705219

**Published:** 2021-07-30

**Authors:** Maryam Shahi, Ricardo Mamber Czeresnia, E. Heidi Cheek, Reade A. Quinton, Rana Chakraborty, Elizabeth Ann L. Enninga

**Affiliations:** ^1^Department of Laboratory Medicine and Pathology, Mayo Clinic College of Medicine, Rochester, MN, United States; ^2^Department of Obstetrics and Gynecology, Mayo Clinic College of Medicine, Rochester, MN, United States; ^3^Department of Immunology, Mayo Clinic College of Medicine, Rochester, MN, United States; ^4^Department of Pediatric and Adolescent Medicine, Mayo Clinic College of Medicine, Rochester, MN, United States

**Keywords:** villitis, cytomegalovirus, placenta, immune tolerance, PD-1, PD-L1, LAG3

## Abstract

Pregnancy is an immunological paradox whereby maternal immunity accepts a genetically unique fetus (or fetuses), while maintaining protective innate and adaptive responses to infectious pathogens. This close contact between the genetically diverse mother and fetus requires numerous mechanisms of immune tolerance initiated by trophoblast cell signals. However, in a placental condition known as villitis of unknown etiology (VUE), there appears to be a breakdown in this tolerance allowing maternal cytotoxic T-cells to traffic into the placenta to destroy fetal villi. VUE is associated with several gestational complications and an increased risk of recurrence in a subsequent pregnancy, making it a significant obstetrical diagnosis. The cause of VUE remains unclear, but dysfunctional signaling through immune checkpoint pathways, which have a critical role in blunting immune responses, may play an important role. Therefore, using placental tissue from normal pregnancy (n=8), VUE (n=8) and cytomegalovirus (CMV) infected placentae (n=4), we aimed to identify differences in programmed cell death 1 (PD-1), programmed death ligand-1 (PD-L1), LAG3 and CTLA4 expression between these etiologies by immunohistochemistry (IHC). Results demonstrated significantly lower expression of PD-L1 on trophoblast cells from VUE placentae compared to control and CMV infection. Additionally, we observed significantly higher counts of PD-1+ (>100 cells/image) and LAG3+ (0-120 cells/image) cells infiltrating into the villi during VUE compared to infection and control. Minimal CTLA4 staining was observed in all placentae, with only a few Hofbauer cells staining positive. Together, this suggests that a loss of tolerance through immune checkpoint signaling may be an important mechanism leading to the activation and trafficking of maternal cells into fetal villi during VUE. Further mechanistic studies are warranted to understand possible allograft rejection more clearly and in developing effective strategies to prevent this condition from occurring *in utero*.

## Introduction

Chronic villitis is a placental condition characterized by inflammation and lymphohistiocytic infiltration into the chorionic villi with or without necrosis, and is categorized into either infectious villitis or non-infectious villitis, also known as villitis of unknown etiology (VUE) (1). While villitis due to infectious etiologies are rare (1-4/1000 pregnancies), 6.6-33.8% of pregnancies are diagnosed with VUE following a term delivery (2–4). Infectious villitis is secondary to transplacental dissemination of microorganisms such as cytomegalovirus (CMV), *Treponema pallidum*, *Toxoplasma gondii* and Herpes simplex viruses (HSV) (5). On the other hand, the diagnosis of VUE requires exclusion of infectious etiologies and is therefore hypothesized to be an anti-fetal allograft response (6, 7). The cellular composition of VUE predominantly includes T-cells and macrophages with minor B-cell involvement (8, 9). Immunohistochemical staining and *in situ* hybridization demonstrate maternal origin of T-cells and both maternal and fetal origin of macrophages, further supporting a breakdown in maternal-fetal tolerance (10, 11).

Pregnancy can be likened to an allograft transplant where the fetus contains a mixture of maternal and paternal genetic material; yet maternal immune responses to the haploidentical fetus during gestation are well-regulated to support the growth and development of offspring. Therefore, the diagnosis of VUE may represent a failure of tolerance and a resulting allograft rejection response to the genetically unique fetus. This hypothesis is supported clinically in that VUE is increased in patients with autoimmune diseases such as systematic lupus erythematosus and thyroid-related disease (12, 13). Neonatal alloimmune thrombocytopenia is also associated with a VUE diagnosis (14). In addition, a higher rate of VUE has been observed in donor oocyte *in vitro* fertilization (IVF) pregnancies, compared to native oocyte IVF, suggesting a higher propensity of immunologic adverse effects in an entirely foreign embryo (15, 16). More recently, pregnancies resulting from IVF had a higher prevalence of VUE diagnoses compared to spontaneously conceived pregnancies (16.2% *vs*. 8.3%) (17). Lastly, VUE recurrence in a subsequent pregnancy is between 10-37%, which suggests that this etiology may lead to the development of a maternal anamnestic memory response, reactivated when re-exposed to the same fetal antigens (5, 18).

The instigator(s) of VUE still remain unknown. A unique feature of the placenta is the expression of non-classical major histocompatibility class I (MHCI) surface receptors, like human leukocyte antigen (HLA)-G, which is uniquely expressed on trophoblasts. These cells come in direct contact with maternal blood, and yet do not elicit immune recognition as antibodies directed at HLA-G are not detected in multigravid women (19, 20). Instead, high expression of HLA-G leads to short term tolerance by binding ILT2 and KIR receptors on numerous immune cells, and the release of soluble isoforms into the plasma throughout pregnancy (21, 22). We note that MHC I/II molecules are not expressed in normal term placentae (with the exception of HLA-C), but can be detected at high levels in placentae diagnosed with VUE (23, 24). Upregulation of MHC I/II on the trophoblast surface of placentae with VUE could promote fetal antigen presentation facilitated by activated macrophages and maternal T-cells (13). Cytokines and chemokines also play a critical role in maternal-fetal tolerance and could have an important role in maintenance of tolerance or establishment of placental inflammation. Chemokines CXCL9, CXCL10 and CXCL11 have been detected in maternal blood and placentae of cases diagnosed with VUE (23, 25). Other mechanisms to maintain tolerance include clonal deletion, FAS/FAS L interactions, indoleamine2,3-dioxygenase (IDO) expression and the presence of T-regulatory cells (26, 27).

Other mechanisms of immune tolerance, such as checkpoint inhibitors, have not been investigated in the setting of VUE. Immune checkpoint inhibitors on T-cells bind to their respective ligands on antigen presenting cells and abrogate immune responses, thereby preventing over-activation of immunity and the development of autoimmune disease. In the past 10 years, the critical role of the PD-1/PD-L1 interaction in tumor immune tolerance has been established, and effective treatments targeted at disrupting this interaction to reactivate an exhausted immune system against the tumor have been developed. PD-1/PD-L1 promotes immune evasion by tumor cells and may be pivotal in maternal-fetal tolerance (28, 29). PD-L1 is expressed by trophoblast cells and circulates in maternal blood during pregnancy (30, 31). Other immune checkpoint molecules include the lymphocyte activation gene protein 3 (LAG3) and cytotoxic T lymphocyte antigen-4 (CTLA4). LAG3 has been described to synergistically act with PD-1/PD-L1 to dampen immune responses by suppressing cytotoxic T-cell signaling (32). Similarly, CTLA4 competes with immune stimulating CD28 on T-cells to bind CD80 and CD86 on antigen presenting cells, inhibiting the proliferation of T-cells and the release of pro-inflammatory IL-2 (33). These semi-redundant pathways have evolved to provide multiple levels of protection against unwarranted immune responses and may have an important role in the loss of fetal-maternal tolerance that likely occurs during VUE.

Therefore, the goal of this study was to evaluate the expression of PD-1, PD-L1, LAG3 and CTLA4 in placentae diagnosed with VUE and compare it to expression in infectious villitis and normal controls. We hypothesized that the expression and abundance of these targets will significantly differ from infectious villitis and controls because of dysregulation of immune tolerance during VUE. To test this hypothesis, tissues from paraffin blocks were cut and immunohistochemistry completed to identity differences in these immune checkpoint molecules.

## Methods

### Case Selection

The Mayo Clinic Institutional Review Board approved this study (#16-006099). The aim of the study was to compare differences in checkpoint inhibitor expression in placentae with VUE, cytomegalovirus (CMV) infection, and controls by immunohistochemistry (IHC). Lymphocytes were identified by H&E and hemotoxylin-counterstained IHC slides based on their cytomorphologic features, including round small nuclei with dense chromatin pattern, inconspicuous nucleoli, and small amount of cytoplasm. Following histologic review by a pathologist, residual formalin fixed paraffin embedded placental tissues were identified. We chose 8 placentae with a diagnosis of high grade VUE per definition by Khong et al. (34) (presence of multiple foci of villitis, on more than one section, at least one of which shows inflammation affecting more than 10 contiguous villi), 4 placentae diagnosed with placental infection by cytomegalovirus (CMV) and 8 gestational age-matched controls to the VUE cases. The control cases had no identifiable pathology.

### Immunohistochemistry (IHC)

Five-micron sections were cut, and tissues underwent deparaffinization by xylene followed by antigen retrieval using citrate buffer. Tissues were then stained with the following primary antibodies overnight at 4°C: PD1 (clone NAT105, 1:200), PDL1 (clone SP142, 1:400), CTLA-4 (clone F-8, 1:50) and LAG3 (clone 11-E3, 1:200). Following secondary antibody incubation for 1 hour at room temp, slides were treated with 3,3′-Diaminobenzidine (DAB, Sigma-Aldrich) for 15 minutes followed by hemoxylin. Images were captured using a CellSens Standard (Olympus Corporation Tokyo, Japan) on a BX51 Olympus microscope (Olympus Corporation Tokyo, Japan).

### IHC Quantification

Measurements of PDL-1 membrane thickness and DAB intensity were made using ImageJ Fiji, following the protocol described by Crowe et al. (35). Ten representative images from each case were gathered, blindly analyzed and compared. For PD-L1 thickness, five separate measurements of villi from each representative image were collected and averaged. Thus, for every case, the mean membrane PDL-1 deposition was obtained by fifty distinct measurements. To calculate DAB intensity, five images from each case measured at a threshold set at 131 for each case. To quantify PD-1, CTLA-4 and LAG3, the slides were reviewed by a pathologist. In scanning power (10X), the hotspot areas were identified and positive cells in 20 villi were counted in high power (400X). If ≤20 villi in the entire section had positive cells, all the positive cells in that sections were counted.

### Data Analysis

Patient demographics are reported as medians with ranges. Results from our three groups were analyzed using Kruskal-Wallis testing controlling for the false discovery rate (FDR) with the method of Benjamini, Krieger and Yekutieli. Significance was defined as a p-value ≤ 0.05. All statistical analyses and graphing were performed using GraphPad Prism software version 9.1 (GraphPad Software, San Diego, CA).

## Results

### Patient Characteristics

Twenty placental specimens were included in this study, 8 with a VUE diagnosis, 4 with a CMV diagnosis and 8 identified to be normal following pathological review. Demographics can be seen in **Table 1**, displayed as medians and ranges. There were no significant differences in our groups based on maternal age (average maternal age of 31 years in our cohort). The number of prior pregnancies was 2.5, which was similar between groups. There was a significant difference in gestational age at birth, with the CMV infected cohort having earlier births compared to controls and VUE cases. The median gestational age at birth in the VUE and control groups was also the similar. All mothers had an uncomplicated postpartum course and the infant APGARS at 1 and 5 minutes were equivalent (p=0.20 and p=0.59, respectively).

**Table 1:** Basic cohort demographics.

* *	*Control (n = 8)*	*VUE (n = 8)*	*CMV (n = 4)*	*P-value*
*Maternal Age (y)*	35 (27-39)	30 (22-38)	25 (24-32)	0.0651
*Gravidity*	2.5 (1-5)	2.5 (1-6)	2 (2-3)	0.8092
*Gestational Age at Birth (weeks.days)*	39.4 (37-40.3)	39.0 (34.4-40.3)	34 (31-36)	0.0082
*Female Fetus (%)*	50	63	60	
*APGAR 1 minute*	7 (5-8)	6 (0-9)	3.5 (0-8)	0.2037
*APGAR 5 minutes*	9 (6-9)	8 (4-9)	7.5 (0-9)	0.5858

Data presented as medians with ranges.

### PD-L1 Is Decreased in Non-Infectious Chronic Villitis

**Figure 1:**
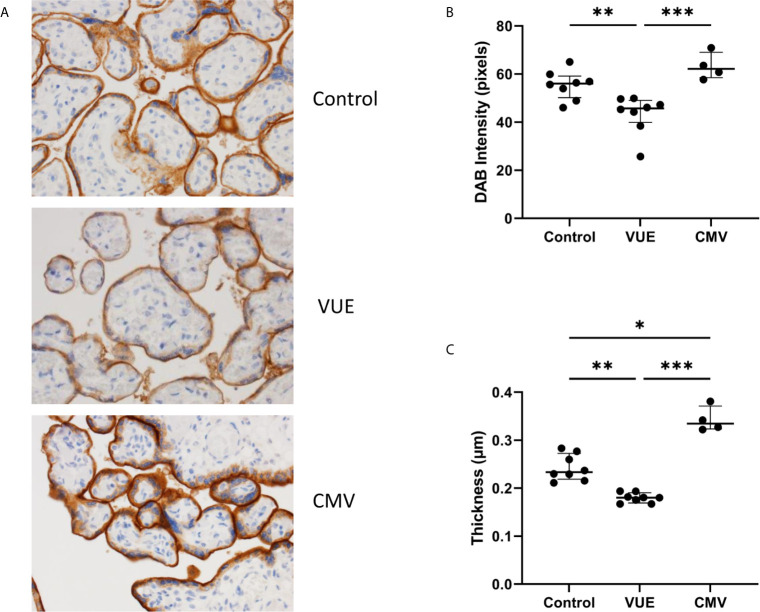
PD-L1 expression in placentae with infectious and non-infectious diagnoses. **(A)** Representative PD-L1 staining in all three groups (200X). **(B)** Average intensity of DAB staining between control, VUE and CMV groups. **(C)** Mean PD-L1 membrane thickness between groups. Graphs show median with interquartile ranges. Data was compared by Kruskal-Wallis testing with *post hoc* analysis using the false discovery rate method of Benjamini, Krieger and Yekutieli (n=4-8/group). *p ≤ 0.05; **p ≤ 0.01; ***p ≤ 0.0001.

To address the potential role of PD-L1 expression during infectious and non-infectious etiologies, we ran IHC and took 10 representative images from each case for analysis using ImageJ. There were observable differences between the groups by microscopy (**Figure 1A**). As others have reported (36), PD-L1 staining was mainly localized to syncytiotrophoblasts, with moderate staining on intermediate trophoblasts and minimal expression on cytotrophoblast cells. We therefore quantified the DAB signal and found that the VUE cases had significantly lower PD-L1 staining intensity compared to control (p=0.009) and CMV placentae (p=0.0008; **Figure 1B**). Although we saw some evidence of increased PD-L1 expression in the CMV group compared to controls, differences between groups did not meet statistical significance (p=0.052). We then measured the thickness of staining across the membrane and note that CMV infection led to the most profuse villous staining of PD-L1 compared to controls (p=0.034) while the thickness was significantly decreased in placentae diagnosed with VUE compared to both control (p=0.004) and CMV (p=0.0001; **Figure 1C**). These data indicate that PD-L1 expression is decreased in placentae with a VUE diagnosis and increased during infection with CMV.

### PD-1+ Cells Are Increased in Villous Tissue During VUE and CMV Infection

**Figure 2:**
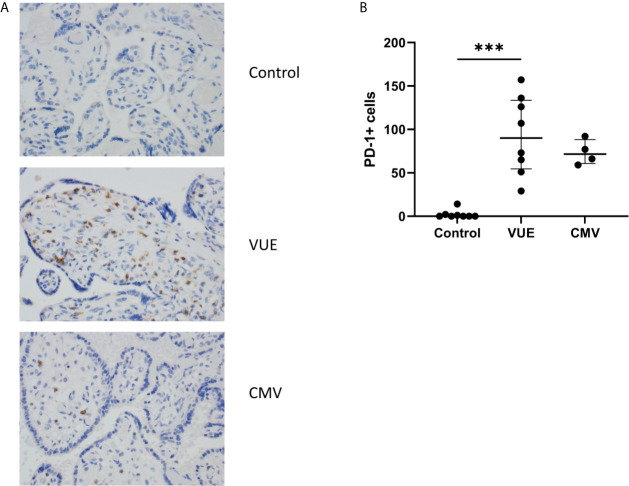
PD-1+ cells in placentae with CMV, VUE and controls. **(A)** Representative PD-1 staining in all three groups (200X). **(B)** Average count of PD-1+ cells per 20 villi in control, VUE and CMV placentae. Graphs show median with interquartile ranges and significance was determined by Kruskal-Wallis testing with false discovery rate correction by Benjamini, Krieger and Yekutieli (n=4-8/group). ***p ≤ 0.0001.

We then quantified PD-1, the receptor to PD-L1, which is expressed on immune cells. Following IHC staining, 10 representative images from each case were captured and counted. Unsurprisingly, there were almost no PD-1+ cells that could be identified in the control placentae (**Figure 2A**). We then counted the number of PD-1+ cells in VUE and CMV. VUE placentae showed the highest number of PD-1+ cells (average 93 cells/image) compared to CMV (73.5 cells/image), which was not a significant difference (p=0.26; **Figure 2B**). Compared to control, non-infectious chronic villitis (p=0.0004) and CMV (p=0.005) had significantly more PD-1+ cells in each image. Thus, PD-1+ cells are most abundant in VUE and CMV infection compared to in normal control tissue.

### Variable Expression of Other Checkpoint Receptors in Placentae

**Figure 3:**
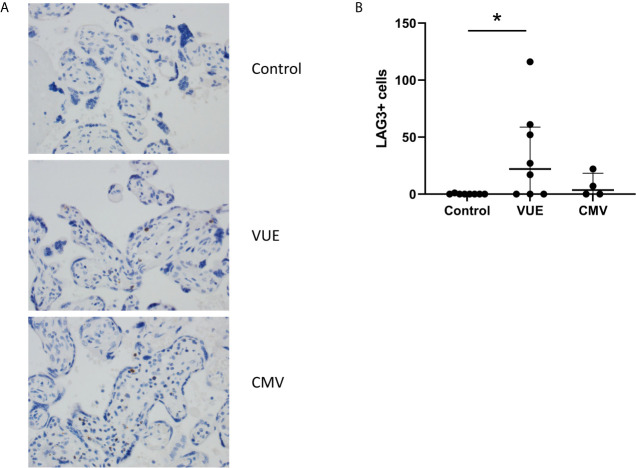
Placental abundance of LAG3+ cells during CMV, VUE and in controls. **(A)** Representative LAG3 staining in all three groups (200X). **(B)** Average count of LAG3+ cells image in each group. Median with interquartile ranges are represented in the graph. Significance was determined by Kruskal-Wallis with false discovery rate correction by Benjamini, Krieger and Yekutieli (n=4-8/group). *p ≤ 0.05.

**Figure 4:**
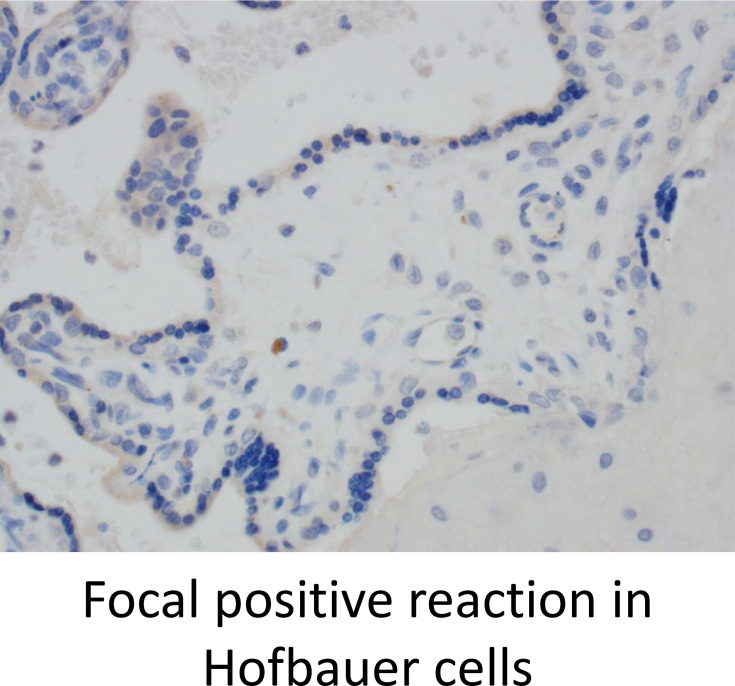
CTLA4 is weakly expressed in the placenta but is found on a few Hofbauer cells. Representative CTLA4 staining in all three groups (200X).

Lastly, we stained for LAG3 and CTLA4, other known checkpoint molecules, in our three groups. Positive cells were counted and averaged from a total of 10 images taken from each case. **Figure 3A** staining demonstrated significant infiltration of LAG3+ cells in VUE compared to controls (49 *vs*. 0.2 cells/image; p=0.05). However, there was a high level of variability noted across the VUE cases (0-116 cells/image; **Figure 3B**). We did not see evidence of a difference in LAG3+ cells between VUE and CMV infection (49 *vs*. 7 cells/image; p=0.399), and CMV infection and control cases (7 *vs*. 0.2 cell/image; p=0.40). In contrast, overall expression of CTLA4+ cells in the placental villi was negative in all three groups, with only a few fetal macrophages staining positive (**Figure 4**). Together, VUE appears to be associated with greater infiltration of LAG3+ cells compared to infection and controls, but cells expressing CTLA4 are generally absent in the placenta from our three groups.

## Discussion

VUE, a diagnosis characterized by the infiltration of maternal CD8+ T-cells into villous tissue of the placenta without infectious cause, is hypothesized to be the result of an immune rejection response targeted against the haploidentical fetus during pregnancy. While the cause remains unknown, we hypothesized that this could be reflect dysregulation of checkpoint molecules which play a critical role in maintaining maternal-fetal tolerance. Our results demonstrate that VUE leads to downregulation of PD-L1 and upregulation of receptors PD-1 and LAG3 on infiltrating cells. This expression pattern is unique from placentae infected with CMV, and the changes in these checkpoint interactions, which ultimately dampen immune responses, may have a significant role in initiation of VUE pathogenesis.

The discovery of proteins that turn off activated lymphocytes, termed checkpoint receptors, has revolutionized the field of tumor immunology through the development of therapeutic strategies that block these interactions, thereby maintaining immune cell activation and tumor cell killing. The first was CTLA4, which binds CD80/CD86 to negatively regulate T-cell activation and blockade leads to anti-tumor immune responses (37, 38). Next, the interaction between PD-1 and PD-L1 was also found to cause cell death in activated T-cells, which tumors expressing high levels of PD-L1 use to evade the immune system (39, 40). Recent focus has turned to disrupting LAG3 signaling through MHC class II to promote immune activation against tumors (41, 42). Like the tumor microenvironment, trophoblasts interact with decidual immune cells at the maternal-fetal interface through checkpoint receptors, which are critical for pregnancy success. Guleria et al. reported increased allogeneic, but not syngeneic, fetal loss in murine models treated with PD-L1 blockade, which also increased proinflammatory cytokine signaling (28). However, other groups have shown that deletion of PD-1 or PD-L1 has no impact on pregnancy outcomes (7). T regulatory cells (T regs) in the normal decidua have been observed to express high levels of CTLA4 and, in specimens from spontaneous abortion cases, the proportion of CTLA4+ T reg cells were found to be significantly lower (43, 44). More recently, upregulation of PD-1 and LAG3 has been found on CD8+ maternal T-cells that recognize fetal antigen from a first pregnancy and are subsequently exposed to the same fetal antigens during a second pregnancy (45). This data proposes a priming mechanism occurs between pregnancies which promotes fetal antigen-specific immune tolerance responses in the mother. Together, these studies indicate a critical role for immune checkpoint receptors in mediating maternal immune tolerance to the allogenic fetus.

Expression of checkpoint receptors in VUE have not been examined, but as this etiology has been hypothesized to be an allograft rejection response, presence or absence of these suppressive regulatory signals are pertinent. In tissue transplant, allograft rejection is a common complication, and occurs when lymphocytes from the recipient infiltrate into donor tissue leading to organ failure (46). Antibody-mediated organ failure, as measured by C4d expression, is used clinically to assess transplant rejection (47). Placentae diagnosed with VUE have been found to have increased staining for C4d in the syncytiotrophoblast layer (48). ICAM-1, a surface glycoprotein that is upregulated upon cytokine stimulation resulting in the migration of immune cells into tissue, is upregulated on trophoblast cells and leukocytes in chronic villitis (49). Interestingly, anti-ICAM-1 antibodies showed preclinical promise for improving allograft survival, randomized clinical trials demonstrated no benefit (50, 51). Demise of a fetus after 20 weeks gestation could be considered an extreme rejection response. In a cohort of 40 fetal demise cases, placental examination showed that approximately 58% had a chronic inflammatory lesion defined as chorioamnionitis, VUE or chronic deciduitis (52). Importantly, chronic inflammation did not correlate with the detection of microorganisms.

Despite the recent advancements in our understanding of VUE as a fetal rejection response, it is not yet possible to rule out the presence of a subclinical infectious etiology. At least in a subset of VUE cases, undiagnosed pathogens were later detected (5, 53). While a bacterial etiology could not be confirmed by PCR for universal bacterial 16S rRNA, at least one herpesvirus strain has been detected in half of the VUE cases tested (54, 55). However, differences in histological and clinical manifestations of VUE from the known infectious etiologies suggest distinct inflammatory processes. Morphologically, VUE tends to involve the placental parenchyma in a patchy pattern with a higher rate of distal villi distribution, whereas in infectious villitis, the distribution is more diffuse with concurrent involvement of umbilical cord, chorionic plate, and membranes (5). While the presence of plasma cells does not exclude a VUE diagnosis, marked plasmacytic infiltration is more often associated with an infectious processes like CMV or other viruses (56).

The results of our study show increased expression of PD-L1 in CMV infectious villitis. CMV infected dendritic cells have been reported to express higher levels of PDL-1 (57). Thus, PD-L1 overexpression by CMV-infected cells could be a mechanism to support viral replication. Having only studied CMV related infectious villitis, we cannot infer that other agents induce similar PD-L1 expression. While much of the focus is on T-cells, immunologic activation in the Hofbauer cells during VUE has also been observed, and studies have demonstrated that CMV has a tropism for Hofbauer cells of the placenta (58, 59). Therefore, Hofbauer cells have an important role in the pathophysiology of both conditions, which requires further characterization. In VUE, our results show increased PD-1 expression in infiltrating lymphocytes and decreased PD-L1 expression in trophoblasts, implying a possible loss suppressive signals leading to the propagation of T-cell activation signals and resulting inflammation. This has been observed in autoimmune disease, where depleting PD-1+ autoreactive T cells in mice with type I diabetes or autoimmune encephalomyelitis led to delayed disease onset and improvement of symptoms (60). Though our small sample size is small, the data demonstrates differences in immune checkpoint receptor expression in infectious versus non-infectious villitis which should be explored further.

To conclude, differential expression of PD-L1 and abundance of PD-1 and LAG3 is seen in placentae diagnosed with VUE compared to CMV infection. These data suggest that disruption of maternal-fetal tolerance through checkpoint receptor signaling may be an important mechanism in the development of VUE. Further understanding of the precise etiology and pathophysiology of VUE will require a multidisciplinary and systems biology approach to effectively address this sometimes-devastating placental condition.

## Data Availability Statement

The raw data supporting the conclusions of this article will be made available by the authors, without undue reservation.

## Ethics Statement

The studies involving human participants were reviewed and approved by Mayo Clinic Institutional Review Board 200 First St. SW Rochester, MN 55905. The patients/participants provided their written informed consent to participate in this study.

## Author Contributions

All authors listed have made a substantial, direct, and intellectual contribution to the work, and approved it for publication.

## Funding

This work was supported by HD065987 (EALE), the Marcia T. Kreyling Career Development Award in Pediatric and Neonatal Research (EALE), the Mayo Clinic Division of Anatomical Pathology (MS) and AI131566, HD097843, HD103498 (RC).

## Conflict of Interest

The authors declare that the research was conducted in the absence of any commercial or financial relationships that could be construed as a potential conflict of interest.

## Publisher’s Note

All claims expressed in this article are solely those of the authors and do not necessarily represent those of their affiliated organizations, or those of the publisher, the editors and the reviewers. Any product that may be evaluated in this article, or claim that may be made by its manufacturer, is not guaranteed or endorsed by the publisher.
